# Recent-Onset Melanoma and the Implications of the Excessive Use of Tanning Devices—Case Report and Review of the Literature

**DOI:** 10.3390/medicina60010187

**Published:** 2024-01-21

**Authors:** Luana-Andreea Nurla, Gina Wafi, Raluca Tatar, Alexandra Maria Dorobanțu, Mădălina Chivu, Liliana Gabriela Popa, Călin Giurcăneanu, Olguța Anca Orzan

**Affiliations:** 1 Faculty of Medicine, ‘Carol Davila’ University of Medicine and Pharmacy, 020021 Bucharest, Romaniaolguta.orzan@umfcd.ro (O.A.O.); 2 Dermatology Clinic, “Elias” Emergency University Hospital, 011461 Bucharest, Romania; 3 Institute of Doctoral Studies, Doctoral School of Medicine, “Ovidius” University of Constanta, 900573 Constanta, Romania; 4 Dermatovenerology Clinic, “Victor Babeș” Clinical Hospital for Infectious and Tropical Diseases, 030303 Bucharest, Romania; 5 Department of Plastic Reconstructive Surgery and Burns, “Grigore Alexandrescu” Clinical Emergency Hospital for Children, “Carol Davila” University of Medicine and Pharmacy, 020021 Bucharest, Romania; 6 Department of Pathology, “Elias” Emergency University Hospital, 011461 Bucharest, Romania

**Keywords:** indoor tanning, melanoma, dysplastic nevi, ultraviolet light exposure, case report

## Abstract

*Introduction*: Melanoma, a malignant tumor arising from uncontrolled melanocytic proliferation, commonly found in the skin but capable of affecting extracutaneous sites, ranks fifth among diagnosed oncological entities and is a significant cause of cancer deaths, constituting over 80% of skin cancer mortality. Genetic factors and ultraviolet radiation (UVR) exposure, from both natural and artificial sources, are the primary risk factors. *Case Presentation*: We reported the case of a 25-year-old female with numerous pigmented nevi and notable changes attributed to extensive indoor tanning sessions. Dermatological examinations and dermoscopic evaluations revealed atypical features in two pigmented nevi, leading to surgical excision. Histopathological and immunohistochemical analyses confirmed a compound nevus in one lesion and superficial spreading melanoma in the other, emphasizing the importance of vigilant follow-up and the correct use of immunohistochemistry. *Discussion*: Indoor tanning significantly elevates the cutaneous melanoma risk, with initiation before age 35 amplifying the risk by up to 75%, especially in young women. The risk escalates with cumulative sessions, particularly exceeding 480, and individuals undergoing over 30 sessions face a 32% higher risk. UVR induces DNA damage, genetic mutations, and immunosuppression, contributing to oncogenesis. Genetic factors, like the PTCHD2 gene, may influence the tanning dependency. Legislation targeting minors has been enacted globally but only with partial efficacy. Tanning accelerators, though associated with minor side effects, correlate with high-risk behaviors. The case underscores the urgency of addressing indoor tanning risks, emphasizing targeted awareness efforts and legislative improvements. *Conclusions*: In conclusion, the reported case highlights the increased risk of cutaneous melanoma linked to indoor tanning, particularly among young women and specific sociodemographic groups. Despite legislative measures, challenges persist, suggesting the potential efficacy of online campaigns involving relatable influencers to raise awareness and discourage artificial tanning.

## 1. Introduction

Melanoma represents a malignant tumor caused by uncontrolled melanocytic proliferation that is most commonly found in the cutaneous organ but may also affect extracutaneous sites, such as the mucous membranes, the eyes, and the leptomeninges. The main subtypes of this oncological pathology comprise superficial spreading melanoma (SSM, 70% of cases), nodular melanoma (NM, 5%), lentigo maligna melanoma (LMM, 4–15%), acral lentiginous melanoma (ALM, 5%), amelanotic melanoma (AM, 4%), and desmoplastic melanoma (DM, <4%) [1].

Melanoma is a major cutaneous malignancy, notably due to its classification as the fifth most diagnosed oncological entity in both sexes and its presence among the top 15 causes of cancer deaths [2], accounting for over 80% of skin cancer mortality [3]. Genetic factors and excessive exposure to ultraviolet radiation (UVR, such as natural light, artificial lighting, or tanning systems) are the main risk factors for the development of skin melanoma [4]. Approximately 80% of skin melanomas develop in cutaneous areas affected by intermittent solar exposure (especially SSMs and NMs), and individuals with personal history of sunburn harbor a twofold increased risk of developing melanoma [5,6]. Furthermore, in-depth studies have shown a greater risk of melanoma occurrence in the adult life of people born in spring, proving the role of the season of birth as a risk factor for increased UVR susceptibility in the perinatal period [7]. 

Ultraviolet radiation (UVR) is classified according to its wavelength in: UVA (315–400 nm), UVB (280–315 nm), and UVC (200–280 nm) [8], while visible light represents the part of the electromagnetic spectrum that the human eye can perceive (400–700 nm) [9]. Despite its beneficial characteristics, consisting in the mediation of vitamin D and endorphin synthesis in the skin [10], UVR is considered a complete carcinogen because of its properties of mutagenesis and tumor initiation and progression [11]. The different detrimental biological effects caused by ultraviolet radiation vary according to its wavelength: UVA is responsible for photoaging, phototoxicity, light-mediated allergic reactions, and the enhancement of the harmful repercussions of UVB, while the latter is involved in the occurrence of erythema due to sunburns and is associated with an augmented risk of skin cancer [12]. 

Apart from the natural solar source of UVR, there are indoor tanning devices that use artificial light and that have been officially labelled as first-group human carcinogens by the International Agency for Research on Cancer of the World Health Organization (WHO) in 2009 [13]. This decision was explained by the higher dose of UVA radiation emitted by devices such as solariums and the quasi-total exposure of the skin to UVA inside a tanning bed, compared to the usual partial cutaneous exposure to natural sunshine [14]. As the major source of non-solar UV exposure, contemporary indoor tanning equipment primarily releases UVA radiation, while only < 5% of the emitted radiation falls within the UVB range, the latter being responsible for promoting a long-lasting tan [15]. The increased negative influence of indoor tanning devices, in comparison with solar light, resides in the emission of radiation 10–15 times more intense than the sunlight on the Mediterranean Sea at 12 PM, in the context of intermittent UV exposure considered uncommon for human skin. 

The present case report aims to highlight the particularities that arise in the nevus pattern, both from a clinical and dermoscopic perspective, after cumulative and consistent exposure to indoor tanning units, and to call attention to the risk of developing melanoma associated with the excessive use of sunbeds. Moreover, the importance of the supplementary immunohistochemical evaluation of morphologically suspicious pigmented lesions that do not exhibit clear-cut diagnostic criteria is emphasized. 

## 2. Case Presentation

A 25-year-old Caucasian female patient, resident in an urban area with over 200,000 inhabitants, with average annual sunshine of 2112 h (latitude 44.439663 and longitude 26.096306), presented in the ambulatory department of the dermatology clinic of an academic hospital, for the recent onset of several pigmented nevi, in the last 12 months, and changes in the appearance of some other pre-existing nevi.

The hereditary collateral history included essential arterial hypertension diagnosed in her mother and diabetes mellitus in her father, with no significant cutaneous pathology detected in any first- or second-degree relative. Moreover, no personal comorbidities were associated; the patient worked as an accountant in an office and, therefore, did not have professional exposure to ultraviolet radiation. The personal sun-damage risk factors included the use of sunbeds 4–5 times per week for about 12 months, with an average duration per tanning session of 7 min and ultraviolet exposure dose of approximately 15 J/cm^2^ per session, corroborated with the application of topical products designed to accelerate the tanning process. The patient totaled over 200 indoor tanning sessions performed with a device that emitted 95% UVA and 5% UVB and stated that she did not follow the indications regarding the exposure position inside the tanning bed (in order to obtain a more intense tan, she placed her cervical extremity where the lower extremities should have been placed and vice versa). No diagnostic challenges related to access to healthcare, cultural, or financial matters were associated with the analyzed case.

**Figure 1:**
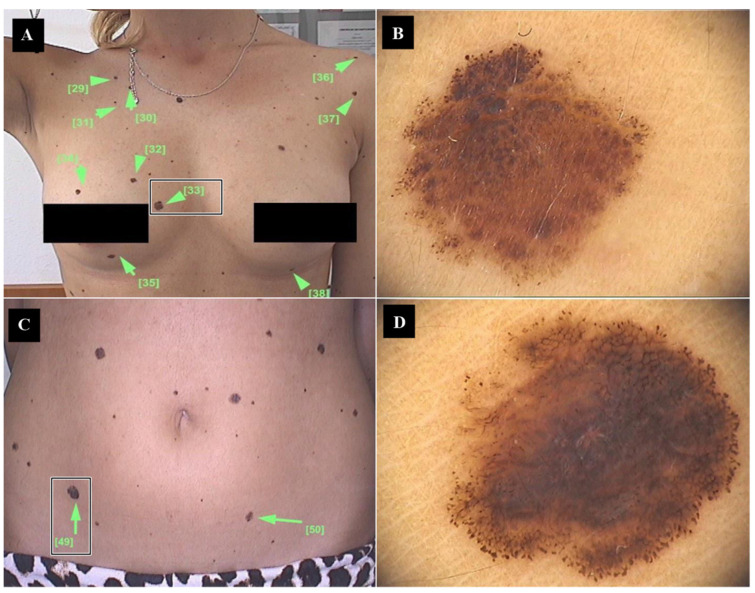
Clinical and dermoscopic aspect of the two suspicious pigmented lesions: (**A**) Lesion no. 33 identified on the right breast; (**B**) dermoscopic image of the cutaneous right mammary melanocytic lesion, showing asymmetry and color variegation; (**C**) Lesion no. 49 identified in the right hypogastric area; (**D**) dermoscopic image of the cutaneous right hypogastric lesion, highlighting an irregular shape, central hyperpigmentation, and peripheral globules.

The dermatological examination revealed current pigmentary characteristics superposable to Fitzpatrick phototype III (Appendix A), due to the cutaneous tan, with a real baseline phototype II, multiple pigmented nevi, with irregular shapes, variable coloration (ranging from light brown to dark brown), and of different diameters. Among over 50 examined melanocytic lesions, two pigmented nevi presented the clinical “ABCD” criteria (asymmetry, irregular borders, multiple colors, diameter > 6 mm). The first atypical pigmentary nevus was located on the right breast (Figure 1A), while the second one was objectified at the level of the right hypogastric region (Figure 1C).

The dermoscopic examination of the first lesion (Figure 1B) revealed global asymmetric morphology, atypical pigment network, variable coloration (light brown, dark brown, and black), with a more evident pigmentation area in the superior left lesional quadrant and peripheral globules; whereas the dermoscopic pattern of the second suspicious lesion comprised irregular shape, asymmetry of color (light brown, dark brown, and black), central hyperpigmentation, and peripheral globules (Figure 1D).

**Figure 2:**
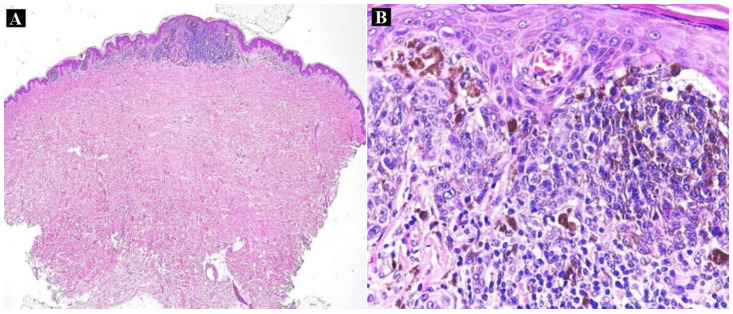
Histopathologic aspect of the right hypogastric lesion: (**A**) asymmetrical melanocytic proliferation with papillary dermal involvement (HE × 4); (**B**) atypical epithelioid melanocytic population with junctional activity, moderate pleomorphism, dusty melanin pigmentation, angiotropism, and atypical mitoses suggestive for superficial spreading melanoma (HE × 20).

Surgical excisional biopsy was performed for both suspicious pigmented lesions, and the histopathological examination stated the diagnosis of compound nevus for the cutaneous right mammary lesion (Figure 2A). On the other hand, the microscopical evaluation of the hematoxylin–eosin (HE)-stained tissue sample excised from the right hypogastric region described features superposable to superficial spreading melanoma, developed on a pre-existing nevus, with a Breslow index (Appendix A) measuring 0.5 mm (Figure 2B).

Furthermore, immunohistochemical (IHC) staining was applied to the fragments obtained from the excised lesions (Table 1), and the immunohistochemical profile identified a compound nevus with dysplastic features or a Clark nevus (Appendix A) associated with abundant inflammatory reaction in the case of the right mammary pigmentary lesion. The central dermal component was surrounded by an abundant lympho-histiocytic infiltrate, similar to that described in a halo nevus (Appendix A); but, it was corroborated with the presence of normal melanocytes in the basal layer of the perilesional tegument. Moreover, preferentially expressed antigen in melanoma (PRAME) was negative in the neoplastic cells, with intense positive external control. Eventually, p16 expression was present in the tumoral proliferation, with a normal checkerboard pattern; while Ki-67 revealed positive reactions in less than 1% of the nuclear population, without proliferation clusters. The proliferative melanocytic nests appeared completely excised on the examined sections. 

**Figure 3:**
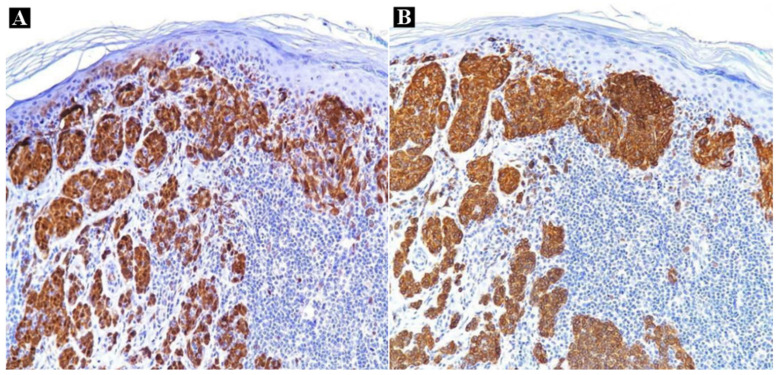
Immunohistochemical aspect of the right hypogastric lesion: intense Melan-A/MART-1 positive reaction with diffuse pattern in an atypical melanocytic population (**A**) Melan-A/MART-1 × 10; (**B**) Melan-A/MART-1 × 20.

Secondly, the immunohistochemical evaluation of the cutaneous right hypogastric lesion generated an intense positive Melan-A reaction, diffusely objectified in the tumoral population, highlighting an asymmetrical silhouette, with the absence of pagetoid epidermal invasion (Figure 3, Appendix A).

**Figure 4:**
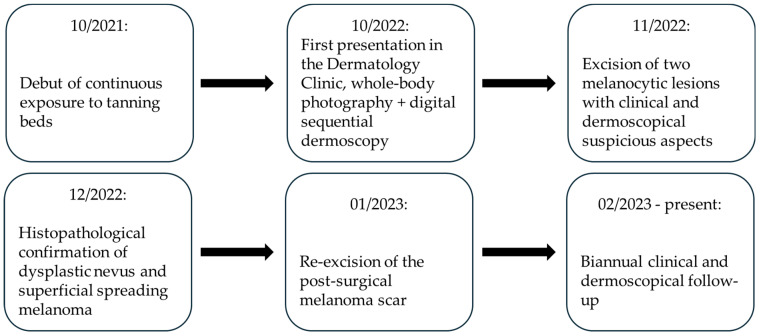
Timeline of the presented medical case.

Moreover, the patient was counseled regarding the discontinuation of tanning bed exposure and advised concerning the avoidance of solar exposure, as well as the application of broad sun protection factor products on the cutaneous areas not covered by clothing. The patient is currently undergoing clinical dermatological exams and digital dermoscopic follow-up at 6-month intervals for the surveillance of the other pigmented lesions (Figure 4).

## 3. Discussion

Exposure to tanning beds is particularly harmful to the skin; studies have shown that the risk of cutaneous melanoma increases with every tanning session, and the first use of tanning beds before the age of 35 leads to an increased risk of cutaneous melanoma of up to 75%, especially among young women [16]. Moreover, a strong association between artificial tanning and the onset of keratinocyte carcinomas (KCs) and melanoma under 40 years of age was proven, a tumor group with a predominance of BRAF mutations and fewer NRAS mutations [17]. 

The research conducted by Gasvand et al. observed an elevated risk of melanoma in women who commenced indoor tanning before the age of 30 and those who started indoor tanning at 30 years of age or older, compared to individuals who were never exposed to sunbeds [18]. Even though engaging in a single indoor tanning session was proven to raise the risk of developing melanoma by 20% [15], it also increased in association with longer durations of use and the cumulative number of indoor tanning sessions; but, it was mainly correlated with the SSM histopathological subtype. The latter was considered particularly harmful when the total number of tanning sessions surpassed 480 sessions per individual, while women with more than 30 artificial tanning sessions experienced a 32% higher risk of melanoma. No variation in the relative risk for melanoma was contingent on the number of sunburns, sunbathing habits, freckling, number of nevi, or hair color. On average, the artificial tanning duration per session ranges between 5 and 20 min; but, it was shown that individuals who commonly use tanning beds spend a significantly longer time tanning every session compared to those who use tanning beds with a lower frequency [19]. 

UV rays cause deoxyribonucleic acid (DNA) damage (formation of pyrimidine cyclobutene dimers), genetic mutations, immunosuppression, oxidative stress, and inflammatory responses, all of which play an important role in photoaging and the skin oncogenetic processes [20], and DNA changes due to UVA rays tend to resolve slower [21].

UV energy influences global homeostasis by being absorbed and transduced into chemical, hormonal, and neural signals in a wavelength-dependent manner, affecting the central nervous system activation and the endocrine glands [22]. This regulation, rooted in evolutionary relics, follows precise neuroendocrine mechanisms, with potential applications in treating autoimmune diseases and mood disorders, underscoring the importance of understanding the UV–skin/eye–brain axis [23]. Furthermore, growing evidence suggests that UV therapy holds potential for treating chemical addiction and mood disorders, attributed to its opioidogenic effects [24]. Additionally, UV may be utilized to influence body metabolism, food intake, and appetite through its impact on pro-opiomelanocortin (POMC), corticotropin-releasing hormone (CRH), and agouti-related protein signaling [25].

In a study by Li et al., light-haired people aged between 25 and 35 were most likely to be associated with indoor tanning habits [26]. Moreover, the notion of the heritable components occurring in alcoholism, nicotine dependence, and pathological illicit-substance use was used to test the hypothesis of a certain gene involvement in tanning dependency (TD) [27]. A recent innovative study examined a panel encompassing more than 300,000 rare and common exomic variants and found a significant association between Patched Domain Containing 2 (PTCHD2), a gene primarily expressed in neural tissues, and TD. The neural origin of this gene could support the existing evidence of a proposed mechanism for TD that implies the expression of UV-induced p53 protein, leading to elevated levels of beta-endorphins and adrenocorticotrophic hormone that occur through the stimulation of the POMC gene promoter [28,29]. 

An Italian study found that the representative individual predisposed to excessive use of sunbeds was characterized by female young adults, as in the case of our patient [30,31], whose most frequent argumentations for solarium use were cosmetic purposes, the erroneous attribution of solarium light as an effective anti-acne therapy [32], the absence of other adequate tanning options (topical products such as sprays and lotions are not considered appealing) [33], and the accessibility and non-prohibitive prices practiced by tanning salons [34]. Moreover, another category of individuals vulnerable to the adoption of indoor tanning behaviors was represented by sexual minorities, such as gay and bisexual men, who have recently been linked to more frequent exposure to indoor tanning devices and higher incidences of melanoma compared with heterosexual men [35].

Taking into account the whole spectrum of the negative effects and consequences of artificial tanning devices, several countries have instituted specific legislation regarding indoor tanning, primarily focused on prohibiting minors’ access in order to safeguard public health matters [36]. France was the pioneer in banning individuals under the age of 18 from using indoor tanning in 1997, a measure later adopted by Brazil in 2002 [37]. Afterwards, various nations followed the example, with some implementing more rigid regulations. In 2011, Brazil extended its ban on indoor tanning access to all age groups, and later, in 2015, Australia prohibited the existence of commercial tanning salons [38]. Enforceable legislation concerning the use of artificial tanning devices, comprising the ban of minors’ access to sunbeds exposure, mandatory parental consent or supervision, compulsory protective eyewear, and the display of warning signs, have been adopted by Australia, the United States, Canada, some European countries such as France and Germany, and South American nations, including Chile [39]. However, mandating parental approval before the use of sunbeds proves unsuccessful, in part because many parents of teenagers who are inclined to use artificial tanning equipment also engage in sunbed usage themselves [40].

Based on the partial compliance to indoor tanning national regulations, either due to the inefficacy of warning messages or the questionable conformity norms adopted by indoor tanning parlors, corroborated with recent findings concerning the high-risk populations, specific social media campaigns have been developed in order to increase the effectiveness of raising awareness regarding the use of artificial tanning beds. Thus, the delivery of preventive messages via social media feeds covering child health topics proved effective and showed the potential to persuade parents to deny permission for indoor tanning, given the fact that mother–daughter similar tanning practices have been established [41]. Furthermore, a study by de Vere Hunt et al. presented evidence demonstrating the feasibility of collaborating with homosexual social media influencers to capitalize on their role as trusted messengers within the high-risk group of sexual minorities prone to excessive sunbed usage that conveyed messages aligning with the objectives of indoor tanning-associated melanoma awareness [42]. 

Tanning accelerators refer to topical products, including but not limited to lotions, oils, and sprays, as well as tablets or injections, employed by individuals engaging in indoor tanning, in order to stimulate melanin production, hence, to enhance and expedite the tanning process [43]. The topical products typically include ingredients such as tyrosine, psoralens, and/or other chemicals that increase photosensitivity and the effects of UV rays. Studies have demonstrated that users of tanning accelerators are often high-risk indoor tanners, who engage in more frequent tanning sessions and display a higher likelihood of tanning addiction. Even though acne and rashes occur more commonly during the use of tanning accelerators, these side effects are not serious enough to determine discontinuation of usage and are, however, minor compared to the potential long-term harmful effects that require supplementary research.

The limitations of the paper firstly reside in the retrospective nature of the study, which poses inherent limitations, as it relies on the accuracy and completeness of historical data, potentially leading to recall bias. Additionally, individual variations in skin type, genetic factors, and exposure to other environmental risk factors are not comprehensively addressed, potentially confounding the observed association. Moreover, the exact dose of UVA and UVB emitted by artificial tanning devices is not properly regulated and displayed in tanning centers; therefore, information on this matter can hardly be standardized. The paper’s focus on a single case raises challenges in generalizing the findings to broader populations, emphasizing the need for larger-scale studies to validate and extend these observations.

The particularities of the reported case reside in the high number of tanning sessions undergone by the patient, the accelerated onset of numerous acquired nevi, the rapid development of cutaneous melanoma after the combination of exposure to indoor tanning equipment and the use of tanning accelerators. In the context of tanning devices and their proven causative association with melanoma, a noteworthy aspect of this study lies in the advocacy for enhanced legislative measures among the Romanian population. Specifically, the proposals include implementing restrictions on power output, diminishing the percentage of UVA emissions in comparison to UVB doses, and setting limits on exposure time per session to a maximum of 5 min, especially in patients with lighter phototypes. This novel perspective emphasizes the dermatologists’ commitment to addressing public health concerns related to artificial UV exposure and may open avenues for broader discussions on regulatory interventions to mitigate the potential risks associated with tanning devices. The consideration of legislative measures underscores the importance of a comprehensive approach to melanoma prevention, encompassing not only awareness campaigns but also regulatory frameworks aimed at minimizing the impact of artificial tanning on skin health.

## 4. Conclusions

In conclusion, this case underscores the heightened risk of cutaneous melanoma linked to indoor tanning, particularly when initiated before age 35, with a notable vulnerability observed in young women. Certain social groups, including young adults, sexual minorities, and individuals with specific cosmetic perceptions, exhibit increased susceptibility to indoor tanning behaviors, and leveraging online campaigns, particularly those involving relatable influencers, emerges as a promising strategy to boost awareness and encourage the discontinuation of artificial tanning. Despite legislative efforts limiting tanning bed access for minors, challenges persist, and this emphasizes the need for a comprehensive approach to melanoma prevention, combining awareness campaigns with regulatory frameworks to minimize the impact of artificial tanning on skin health.

## Figures and Tables

**Table 1:** Immunohistochemical panel.

Immunohistochemical Antibody	Clone	Manufacturer	Dilution	Host, Clonality
PRAME	EPR20330	Biocare	Ready-to-use (RTU) 6 mL	Rabbit, monoclonal
p16	MX007	Master Diagnostica	RTU 3 mL	Mouse, monoclonal
Ki-67	SP6	Master Diagnostica	RTU 7 mL	Rabbit, monoclonal
Melan-A/MART-1	A103	Biocare	RTU 6 mL	Mouse, monoclonal

## Data Availability

The data generated in the present study are included in the figures of this article.

## Appendix A. Short Explanation of Specialty Terminology

Fitzpatrick phototype	Classification of skin types according to the reaction to ultraviolet exposure
Breslow index	Maximal thickness of the malignant melanocytic tumoral population
Clark nevus	Dysplastic or atypical nevus
Halo nevus	Nevus surrounded by a whitish halo (ring)
Pagetoid epidermal invasion	Upward extension of cells (e.g., atypical melanocytes in the case of melanoma) towards the epidermis

## References

[1] Ward W.H., Lambreton F., Goel N., Yu J.Q., Farma J.M., Ward W.H., Farma J.M. (2017). Clinical Presentation and Staging of Melanoma. Cutaneous Melanoma: Etiology and Therapy [Internet].

[2] ECIS—European Cancer Information System Estimates of Cancer Incidence and Mortality in 2022, for All Countries. https://ecis.jrc.ec.europa.eu.

[3] Saginala K., Barsouk A., Aluru J.S., Rawla P., Barsouk A. (2021). Epidemiology of Melanoma. Med. Sci..

[4] Kozmin S., Slezak G., Reynaud-Angelin A., Elie C., de Rycke Y., Boiteux S., Sage E. (2005). UVA radiation is highly mutagenic in cells that are unable to repair 7,8-dihydro-8-oxoguanine in Saccharomyces cerevisiae. Proc. Natl. Acad. Sci. USA.

[5] Gandini S., Autier P., Boniol M. (2011). Reviews on sun exposure and artificial light and melanoma. Prog. Biophys. Mol. Biol..

[6] Arisi M., Zane C., Caravello S., Rovati C., Zanca A., Venturini M., Calzavara-Pinton P. (2018). Sun Exposure and Melanoma, Certainties and Weaknesses of the Present Knowledge. Front. Med..

[7] Crump C., Sundquist K., Sieh W., Winkleby M.A., Sundquist J. (2014). Season of birth and other perinatal risk factors for melanoma. Int. J. Epidemiol..

[8] WHO—World Health Organization Ultraviolet Radiation. https://www.who.int/health-topics/ultraviolet-radiation#tab=tab_1.

[9] Sliney D. (2016). What is light? The visible spectrum and beyond. Eye.

[10] D’Orazio J., Jarrett S., Amaro-Ortiz A., Scott T. (2013). UV radiation and the skin. Int. J. Mol. Sci..

[11] Hamouda S., Alshawish N., Abdalla Y., Ibrahim M. (2022). Ultraviolet radiation: Health Risks and Benefits. Saudi J. Eng. Technol..

[12] WHO—World Health Organization Radiation: Ultraviolet (UV) Radiation and Skin Cancer. https://www.who.int/news-room/questions-and-answers/item/radiation-ultraviolet-(uv)-radiation-and-skin-cancer.

[13] IARC Working Group on the Evaluation of Carcinogenic Risks to Humans (2012). IARC Monographs on the Evaluation of Carcinogenic Risks to Humans Series.

[14] Nilsen L.T., Hannevik M., Veierod M.B. (2016). Ultraviolet exposure from indoor tanning devices: A systematic review. Br. J. Derm..

[15] Boniol M., Autier P., Boyle P., Gandini S. (2012). Cutaneous melanoma attributable to sunbed use: Systematic review and meta-analysis. BMJ..

[16] International Agency for Research on Cancer Working Group on artificial ultraviolet (UV) light and skin cancer (2007). The association of use of sunbeds with cutaneous malignant melanoma and other skin cancers: A systematic review. Int. J. Cancer..

[17] van der Kooij M.K., Wetzels M.J., Aarts M.J., Berkmortel F.W.v.D., Blank C.U., Boers-Sonderen M.J., Dierselhuis M.P., de Groot J.W.B., Hospers G.A., Piersma D. (2020). Age Does Matter in Adolescents and Young Adults versus Older Adults with Advanced Melanoma; A National Cohort Study Comparing Tumor Characteristics, Treatment Pattern, Toxicity and Response. Cancers.

[18] Ghiasvand R., Rueegg C.S., Weiderpass E., Green A.C., Lund E., Veierød M.B. (2017). Indoor Tanning and Melanoma Risk: Long-Term Evidence from a Prospective Population-Based Cohort Study. Am. J. Epidemiol..

[19] Börner F.U., Schütz H., Wiedemann P. (2009). A population-based survey on tanning bed use in Germany. BMC Dermatol..

[20] Meeran S.M., Punathil T., Katiyar S.K. (2008). IL-12 deficiency exacerbates inflammatory responses in UV-irradiated skin and skin tumors. J. Investig. Dermatol..

[21] Khan A.Q., Travers J.B., Kemp M.G. (2018). Roles of UVA radiation and DNA damage responses in melanoma pathogenesis. Environ. Mol. Mutagen..

[22] Slominski A.T., Zmijewski M.A., Plonka P.M., Szaflarski J.P., Paus R. (2018). How UV Light Touches the Brain and Endocrine System Through Skin, and Why. Endocrinology.

[23] Hiramoto K., Jikumaru M., Yamate Y., Sato E.F., Inoue M. (2009). Ultraviolet A irradiation of the eye induces immunomodulation of skin and intestine in mice via hypothalomo-pituitary-adrenal pathways. Arch Dermatol. Res..

[24] Fell G.L., Robinson K.C., Mao J., Woolf C.J., Fisher D.E. (2014). Skin β-endorphin mediates addiction to UV light. Cell.

[25] Slominski A.T. (2015). Ultraviolet radiation (UVR) activates central neuro-endocrine-immune system. Photodermatol. Photoimmunol. Photomed..

[26] Li W.Q., Cho E., Han J., Wu S., Qureshi A.A. (2017). Pigmentary traits and use of indoor tanning beds in a cohort of women. Br. J. Dermatol..

[27] Cartmel B., Dewan A., Ferrucci L.M., Gelernter J., Stapleton J., Leffell D.J., Mayne S.T., Bale A.E. (2014). Novel gene identified in an exome-wide association study of tanning dependence. Exp. Dermatol..

[28] Fisher D.E., James W.D. (2010). Indoor tanning–science, behavior, and policy. N. Engl. J. Med..

[29] Kaur M., Liguori A., Fleischer A.B., Feldman S.R. (2006). Plasma beta-endorphin levels in frequent and infrequent tanners before and after ultraviolet and non-ultraviolet stimuli. J. Am. Acad. Dermatol..

[30] Mastroeni S., Sampogna F., Salcedo N.M., Ricci F., Fania L., Antonelli F., Abeni D., Cristofolini M. (2021). Factors associated with sunbed use among 3692 outpatients in 18 centers of the Italian Cancer League (LILT). Sci. Rep..

[31] Coups E.J., Phillips L.A. (2011). A more systematic review of correlates of indoor tanning. J. Eur. Acad. Derm. Venereol..

[32] Bali R., Ji-Xu A., Felton S.J. (2022). The significant health threat from sunbed use as a self treatment in patients with acne. Clin. Exp. Dermatol..

[33] Lyons S., Lorigan P., Green A.C., Ferguson A., Epton T. (2021). Reasons for indoor tanning use and the acceptability of alternatives: A qualitative study. Soc. Sci. Med..

[34] Glanz K., Jordan A., Lazovich D., Bleakley A. (2019). Frequent Indoor Tanners’ Beliefs About Indoor Tanning and Cessation. Am. J. Health Promot..

[35] Mansh M., Arron S.T. (2016). Indoor tanning and melanoma: Are gay and bisexual men more at risk?. Melanoma Manag..

[36] Reimann J., McWhirter J.E., Papadopoulos A., Dewey C. (2018). A systematic review of compliance with indoor tanning legislation. BMC Public Health..

[37] Pawlak M.T., Bui M., Amir M., Burkhardt D.L., Chen A.K., Dellavalle R.P. (2012). Legislation restricting access to indoor tanning throughout the world. Arch. Dermatol..

[38] Sinclair C.A., Makin J.K., Tang A., Brozek I., Rock V. (2014). The role of public health advocacy in achieving an outright ban on commercial tanning beds in Australia. Am. J. Public. Health..

[39] National Conference of State Legislatures (2018). Indoor Tanning Restrictions for Minors: A State-by-State Comparison. http://www.ncsl.org/research/health/indoor-tanning-restrictions.aspx.

[40] Mayer J.A., Woodruff S.I., Slymen D.J., Sallis J.F., Forster J.L., Clapp E.J., Hoerster K.D., Pichon L.C., Weeks J.R., Belch G.E. (2011). Adolescents’ use of indoor tanning: A large-scale evaluation of psychosocial, environmental, and policy-level correlates. Am. J. Public Health.

[41] Buller D.B., Pagoto S., Baker K., Walkosz B.J., Hillhouse J., Henry K.L., Berteletti J., Bibeau J. (2021). Results of a social media campaign to prevent indoor tanning by teens: A randomized controlled trial. Prev. Med. Rep..

[42] Hunt I.d.V., Cai Z.R., Nava V., Admassu N.E., Bousheri S., Johnson T., Tomz A., Thompson J., Zhang L., Pagoto S. (2023). A Social Media-Based Public Health Campaign to Reduce Indoor Tanning in High-Risk Populations. AJPM Focus..

[43] Herrmann J.L., Cunningham R., Cantor A., Elewski B.E., Elmets C.A. (2015). Tanning accelerators: Prevalence, predictors of use, and adverse effects. J. Am. Acad. Dermatol..

